# Poster Session II - A304 CHARCATERIZATION OF RESPONSE PHENOTYPES FOR MOLECULAR TARGETED THERAPIES IN CROHN’S DISEASE

**DOI:** 10.1093/jcag/gwaf042.304

**Published:** 2026-02-13

**Authors:** E Fortin, A Simard, G Langevin, A Lavoie, A Michaud-Herbst, K Tremblay

**Affiliations:** Pharmacologie-physiologie, Universite de Sherbrooke, Sherbrooke, QC, Canada; Centre integre universitaire de sante et de services sociaux du Saguenay-Lac-Saint-Jean du Quebec, Chicoutimi, QC, Canada; Centre integre universitaire de sante et de services sociaux du Saguenay-Lac-Saint-Jean du Quebec, Chicoutimi, QC, Canada; Centre integre universitaire de sante et de services sociaux du Saguenay-Lac-Saint-Jean du Quebec, Chicoutimi, QC, Canada; Centre integre universitaire de sante et de services sociaux du Saguenay-Lac-Saint-Jean du Quebec, Chicoutimi, QC, Canada; Pharmacologie-physiologie, Universite de Sherbrooke, Sherbrooke, QC, Canada

## Abstract

**Background:**

In Canada, approximately 160,000 persons suffer from Crohn’s disease (CD). For moderate to severe CD, molecular targeted therapies (MTT) are used. Despite their cost, ∼30% of patients show no response. Furthermore, they are the last line therapy before surgery. In this context, response predictive markers are needed. Although pharmacogenetic variants associated with MTT response are reported, none are used in clinic to optimize response to MTT. A literature review revealed that no standard definition exists in the assessment of drug response in the treatment of CD . This heterogeneity in response assessment limits the robustness of pharmacogenetic studies, their replication and the generalization of their results. We hypothesize that the assessment of response phenotype to MTTs in the treatment of CD is possible using retrospective data available in patients’ medical records and could be a tool for phenotype characterization.

**Aims:**

1) To document the use of MTTs in CD patients in a tertiary referral hospital. 2) To phenotype response to MTTs in CD patients.

**Methods:**

This project is a retrospective observational study based on the review of medical records from all adult CD patients who used at least one MTT (infliximab, adalimumab, vedolizumab, ustekinumab, upadacitinib, risankizumab) at the CIUSSS-SLSJ. The records from 2014 to 2025 are available digitally, while those prior to 2014 are in paper format (hospital archives). A gastroenterologist and a pharmacist were involved in creating an initial phenotyping decision tree.

**Results:**

The full cohort has 650 CD patients. From the 100 records reviewed so far, 86 were included in these interim results (53 were female, median age of 26 at diagnosis). Adalimumab (ADA;39), ustekinumab (UST;33) and infliximab (INF;28) were the most used MTTs and 91.8% of the patients used two or less MTT. Using the preliminary criteria, 37.5% of INF users, 57.9% of ADA users and 58.6% of UST users were considered responders. For INF (20.8%) and ADA (21.1%) a fifth were phenotyped as non-responders while UST had 13.8% of non-response. Intermediate response phenotypes were found in 10% of ADA and UST users as well as 25% of INF users. The results will be updated for the presentation.

**Conclusions:**

Our interim results show the feasibility to retrospectively phenotype the response to MTTs in CD. Our criteria will refine and ultimately yield a proposed definition to optimize MTT response phenotyping, aiding future pharmacogenetic studies.

**Funding Agencies:**

Réseau Québecois de Recherche sur le Médicament, Université de Sherbrooke

